# Chloroform, a New Anæsthetic Agent

**Published:** 1848-01

**Authors:** 


*Chloroform, a New Anæsthetic Agent.*—As the readers of the Journal are all aware, the idea of preventing pain in surgical operations, by the inhalation of a volatile agent, originated with, or rather, its practicability was first fully demonstrated by Dr. Morton of Boston, and the substitution by another of any less objectionable and more efficient agent, than that which he employed, will not, in the least, dim the glory which he has won by the discovery, nor pluck a single leaf from the laurels which he has richly earned, should it ultimately prove to be as valuable as it is at present thought to be, by many eminent surgeons both in Europe and America.

For so simple an operation as the extraction of a tooth we have never been a very warm advocate for the inhalation of the vapor of sulphuric ether, the anæsthetic agent hitherto employed, for the purpose of producing insensibility. The injurious effects liable to result from it, as well as its disagreeable and offensive odor, have constituted with us very serious objections to its use in so simple an operation. These objections, however, have been removed by Professor Simpson of Edinburgh, in the substitution of chloroform, or the perchloride of formyle for ether, which he has found by experiment to be a more powerful anæsthetic agent. A very interesting paper on its properties and effects, read by Professor S. before the Medico-Chirurgical Society of Edinburgh, on the tenth of Nov. 1847, will be found in another part of the present number of the Journal, and for this early opportunity of laying it before our readers, we are indebted to Mr. Spence, an eminent dentist of that place, who sent it to us on the day of its publication. In a note accompanying the pamphlet, Mr. Spence speaks of it as being likely altogether to supersede the use of ether, as "it is more easily administered, more rapid in its effects, more agreeable to the patient and more certain in producing the anæsthetic effect."

We have as yet had an opportunity of administering it to only a few, and, therefore, are not prepared to express our opinion upon the subject as fully as we may hereafter be able to do, after we shall have more thoroughly tested its effects. Thus far, we have exercised much caution in administering it, but from the few experiments which we have made, we are inclined to believe that it is far superior to ether. It is certainly more pleasant to the patient—the odor of it not being in the least disagreeable, and it is more efficient and rapid in producing the anæsthetic effect. So far as our own observations go upon the subject none other than the most delightful sensations are produced by its inhalation, and it leaves no disagreeable effects about the head, nausea, or other unpleasant symptoms. The inhalation of ether, at first excites the action of the heart and arteries, and accelerates the pulse, the anæsthetic, seeming to be a secondary effect. Chloroform does not, or at any rate, in so manifest a degree; it apparently produces an immediate sedative effect without materially increasing the frequency of the pulse.

We have been assisted, in all the experiments which we have made with the chloroform, by Professor T. E. and Dr. J. Bond. The last named gentleman has given us the following brief statement of the results.

"The first person to whom it was administered, was Mr. A., a student of dentistry, who had frequently been under the influence of sulphuric ether, and inhaled the chloroform for the purpose of comparing the effect of the latter with the former. He did not desire an operation. The effect upon this gentleman was decisive of the virtue of the agent in the production of total insensibility. After inhaling the vapor for forty seconds, he became totally unconscious, in which state he remained sufficiently long to have had a tooth extracted. Mr. A. decidedly prefers the chloroform to the ether as being more pleasant to inhale, more speedy in its effects, and leaving no unpleasant sensation after the restoration of consciousness. The next subject was a gentleman who had also been under the influence of sulphuric ether a number of times. In this case the chloroform was inhaled for one minute, when it produced total insensibility. While under the influence of the agent a lower bicuspis was extracted without the slightest pain to the patient. I would mention that in order to extract the tooth, the operator, Dr. Cone, was obliged to cut away the alveolar process surrounding it; thus rendering the operation more tedious than usual, and affording a better test. Several other persons submitted themselves to the experiment without testing the effect by an operation, but some of them might have undergone similar operations without suffering any pain. One gentleman complained afterwards of a slight tinnitus aurium, which passed off in a few minutes. The next person who took it had two upper molares extracted by Dr. Noyes, which were so firmly adherent to their alveoli, that a considerable portion of the sockets came away with the teeth. From the extraction of the first tooth the patient suffered no uneasiness, though he afterwards said he was mentally conscious of the operation. During the extraction of the second he was in a profound snoring sleep, which lasted some seconds after the operation. The same patient has since had two more extracted; but the effect before the removal of one had nearly passed off before the operation was performed, consequently it caused some pain. In all these cases the vapor was administered by means of Dr. Roper's inhaler. The effect of the chloroform appears to be directly sedative, and productive of no unpleasant effect upon the brain."

Since writing the above, the same patient has had three lower molar teeth extracted, while under its influence, without suffering any pain.

For the early opportunity we have had of testing the effects of chloroform, we are indebted to Dr. Andrews of Baltimore, one of our most scientific and best practical chemists, who immediately on application, prepared it for us.

In conclusion, we do not wish to be understood as recommending the use of even chloroform in so simple an operation as the extraction of a tooth. We have not yet witnessed enough of its effects to assure ourself that it is perfectly safe. We shall, however, keep our readers advised of the results of such experiments as may be made with, as shall come to our knowledge.

Since the above was written, the Baltimore American has copied from a late Liverdool paper, the following report of surgical operations performed upon individuals while under the anæsthetic influence of chloroform.—*Balt. Ed.*

*The New Agent for producing insensibility of pain.*—In pursuance of an invitation given by Hugh Neill, Esq., the surgeon of the Eye and Ear Infirmary in this town, several gentlemen, eminent in the medical profession, attended on Tuesday last, at the Ophthalmic Institution, to witness some operations for squinting, upon patients while under the influence of "chloroform," a new agent, said to produce complete unconsciousness, more easily and more efficiently than sulphuric ether. The new agent is very pleasant to the palate, and yields a delightful perfume, not much unlike that of a ripe apple, or good sherry wine. Its inhalation produces the most delightful sensations, and, in the course of a minute or so, a state of the soundest somnambulism. Sulphuric ether, on the contrary, is very disagreeble both to the palate and the olfactory nerves, and is much longer in producing the required result. It also produces great nausea, and has not always been administered without acting for a time somewhat injuriously upon the nervous system.

Among the medical gentlemen present at the Ophthalmic Institution, on Tuesday, were Dr. Formby, Dr. Sutherland, the talented editor of The Health of Town's Journal; Dr. Imlach, Mr. Waldie, Mr. Higginson, Mr. Bickersteth, Dr. Hunter Robinson, of Birkenhead, author of a pamphlet on the sanitary state of that township; and many gentlemen connected with the hospitals and dispensaries of Liverpool.

Mr. Neil, previous to operating upon either of the patients, gave a short history of the discovery of ether as an agent to produce insensibility to pain, and, lastly, of "chloroform," as a more pleasant and satisfactory substitute. In the course of his remarks he noticed curious facts of the chloric ether having been first used, and recommended to Professor Simpson by Dr. Formby, and chloroform having been also recommended to the same gentlemen by Mr. Waldie, chemist of the Liverpool Apothecaries' Company, who pointed out to the professor the advantages to be derived from its use.

The patients selected on this occasion were four young females, the only blemish to whose round and handsome faces was that they had each rather more than "an interesting cast of the eye"—in fact that they squinted very badly. The operation whereby this affliction can be totally remedied is necessarily a painful one, though happily one of very short duration, or it would, perhaps, be unbearable by any person acted upon in the natural state.

The chloroform was administered in the three first cases, through the medium of a handkerchief, held up to the nasal organ and mouth, and, as insensibility was produced, the patients were operated upon by Mr. Neill, in the most successful manner; but the fourth case furnished a triumphant instance of the power of the new agent, not perhaps that the other cases would not have been equal to it if the assistants had been used to their work. With the experience furnished by them, the requisites necessary to a most complete administration of the chloroform, by means of the sponge, were systematically and noiselessly executed, and the patient was speedily thrown into a state of complete unconsciousness. During the inhalation of the vapor through the sponge, which of course allows a free passage to the atmospheric air, her countenance wore a most pleasurable expression ; nor did it alter in the least during the trying moments of the operation. She remained in a sound slumber for several moments afterwards and did not evince the slightest sensation of feeling when repeatedly pinched by many of the gentlemen present. In this case, the eye was turned so completely inwards, that greater force with the forceps was required, and had the patient been operated upon in her natural state, the pain would have been proportionably excruciating. Upon waking to consciousness in about a quarter of an hour, she said she was not aware that anything at all had been done to her. The medical gentlemen present thought this case furnished a most excellent test of the efficacy of the new agent, and a vote of thanks was unanimously passed to Mr. Neill by the gentlemen present, for the opportunity he had afforded them of witnessing these interesting experiments.

All the four patients were thus completely cured of obliquity of vision, and being out-patients, were enabled to walk to their respective homes in an hour or two after the operation had been performed. Upon inquiry, in the couse of the afternoon, Mr. Neill found them all in excellent health, and thankful for the service that had been rendered to them.

*Experiments with Chloroform.*—Several important and most painful operations were performed on Thursday, at the Workhouse Hospital, by Dr. F. H. Brett, in the presence of the staff, and other gentlemen, when the new agent for producing insensibility was administered with entire success in the following cases.

The first was the case of Elizabeth Birch, aged 22, affected for years with inversion of the eyelids (entropion) causing inflammation of the eye by the constant irritation of the lashes against the globe. It was necessary to make perpendicular incision at the outer angle of the lids, and to excise a portion of the skin of the upper eyelid, to introduce a suture, and partially to evert the lid by means of a ligature passed through the margin of the lid, this last being secured on the forehead by strips of adhesive plaster. The patient was in a state of perfect insensibility the whole of the time.

The second was a case of strabismus.

The third a hydrocele, which after tapping, was followed by injection of the sack, with a solution of iodine.

The fourth operation was for cataract, which being of a soft nature, the drilling method through the cornea by means of a delicate needle, was selected, the fluid portion of the soft lenticular body being freely exposed to solution in the aqueous humor.

The fifth case was that of dividing the tendons behind the knee-joint (the ham strings) in both legs of Bridget Mackle. The woman had been a cripple for three years, bent double, and bed-ridden—the legs are now straight, and are to be kept on splints for a short time.

The sixth case was that of a young woman, named Elizabeth Felliwell, who had been discharged from the hospital as incurable, from recto vaginal communication, complicated with several tumors deeply situated. The parts being freely exposed by the speculum, a clean wound was made of the former and the morbid growth completely extirpated. No operation could well be conceived more painful than this under ordinary circumstances, yet the poor girl was taken to her bed, in a perfect trance, and was quite unconscious of any part of the operation.

The 7th case was precisely of a similar nature in a young woman named Ann M'Ney. She had been much debilitated, but under remedies to improve her constitutional power, nourishing diet and wine, she was sufficiently restored in health to undergo the operation. The chloroform, however, happily saved her from any knowledge of the operation. She awoke complaining bitterly that she was not to undergo the operation which she had been so long promised was to relieve her from a complaint, the most distressing to which a female can be subjected, until she was assured that all was over.

The eighth, and last, was a case of scrofulous disease of the knee-joint, in which it was necessary to remove the limb in a girl, aged six years. This child knew nothing of what was going to be done—the chloroform kept her in utter insensibility of what was done, and she only awoke to be conscious of the absence of a limb, the seat of protracted disease, which had nearly exhausted her vital powers.

Doctor Burgess administered the chloroform, merely applying it on lint to the mouth and nostrils, whilst Drs. Gee, Bride, and Mr. J. Ferguson, with their usual tact and humanity, rendered every requisite assistance.

The patient, on coming to, seemed to be excited in a manner not dissimilar from the effects of an overdose of champagne.

